# The Combination of Curaxin CBL0137 and Histone Deacetylase Inhibitor Panobinostat Delays KMT2A-Rearranged Leukemia Progression

**DOI:** 10.3389/fonc.2022.863329

**Published:** 2022-05-23

**Authors:** Lin Xiao, Mawar Karsa, Emma Ronca, Angelika Bongers, Angelika Kosciolek, Ali El-Ayoubi, Jezrael L. Revalde, Janith A. Seneviratne, Belamy B. Cheung, Laurence C. Cheung, Rishi S. Kotecha, Andrea Newbold, Stefan Bjelosevic, Greg M. Arndt, Richard B. Lock, Ricky W. Johnstone, Andrei V. Gudkov, Katerina V. Gurova, Michelle Haber, Murray D. Norris, Michelle J. Henderson, Klaartje Somers

**Affiliations:** ^1^ Children’s Cancer Institute, Lowy Cancer Research Institute, University of New South Wales, Randwick, NSW, Australia; ^2^ School of Women’s and Children’s Health, University of New South Wales, Randwick, NSW, Australia; ^3^ Australian Cancer Research Foundation (ACRF) Drug Discovery Centre for Childhood Cancer, Children’s Cancer Institute, Lowy Cancer Research Centre, University of New South Wales (UNSW) Sydney, Sydney, NSW, Australia; ^4^ Leukaemia Translational Research Laboratory, Telethon Kids Cancer Centre, Telethon Kids Institute, Perth, WA, Australia; ^5^ Curtin Medical School, Curtin University, Perth, WA, Australia; ^6^ Curtin Health Innovation Research Institute, Curtin University, Perth, WA, Australia; ^7^ Department of Clinical Haematology, Oncology, Blood and Marrow Transplantation, Perth Children’s Hospital, Perth, WA, Australia; ^8^ Division of Paediatrics, School of Medicine, University of Western Australia, Perth, WA, Australia; ^9^ Gene Regulation Laboratory, Cancer Research Division, Peter MacCallum Cancer Centre, Melbourne, VIC, Australia; ^10^ The Sir Peter MacCallum Department of Oncology, The University of Melbourne, Melbourne, VIC, Australia; ^11^ Department of Cell Stress Biology, Roswell Park Cancer Institute, Elm and Carlton Streets, Buffalo, NY, United States; ^12^ University of New South Wales Centre for Childhood Cancer Research, Sydney, NSW, Australia

**Keywords:** KMT2A-rearranged leukemia, infant leukemia, curaxin CBL0137, histone deacetylase inhibition, chromatin

## Abstract

Rearrangements of the *Mixed Lineage Leukemia* (*MLL/KMT2A*) gene are present in approximately 10% of acute leukemias and characteristically define disease with poor outcome. Driven by the unmet need to develop better therapies for KMT2A-rearranged leukemia, we previously discovered that the novel anti-cancer agent, curaxin CBL0137, induces decondensation of chromatin in cancer cells, delays leukemia progression and potentiates standard of care chemotherapies in preclinical KMT2A-rearranged leukemia models. Based on the promising potential of histone deacetylase (HDAC) inhibitors as targeted anti-cancer agents for KMT2A-rearranged leukemia and the fact that HDAC inhibitors also decondense chromatin *via* an alternate mechanism, we investigated whether CBL0137 could potentiate the efficacy of the HDAC inhibitor panobinostat in KMT2A-rearranged leukemia models. The combination of CBL0137 and panobinostat rapidly killed KMT2A-rearranged leukemia cells by apoptosis and significantly delayed leukemia progression and extended survival in an aggressive model of MLL-AF9 (*KMT2A:MLLT3*) driven murine acute myeloid leukemia. The drug combination also exerted a strong anti-leukemia response in a rapidly progressing xenograft model derived from an infant with KMT2A-rearranged acute lymphoblastic leukemia, significantly extending survival compared to either monotherapy. The therapeutic enhancement between CBL0137 and panobinostat in KMT2A-r leukemia cells does not appear to be mediated through cooperative effects of the drugs on *KMT2A* rearrangement-associated histone modifications. Our data has identified the CBL0137/panobinostat combination as a potential novel targeted therapeutic approach to improve outcome for KMT2A-rearranged leukemia.

## Introduction

Approximately 10% of leukemias harbor a rearrangement of the *Mixed Lineage Leukemia* (*MLL*) gene, now renamed *KMT2A* [reviewed in (1)]. The prevalence of *KMT2A* gene rearrangements is bimodal with translocations found in up to 80% of infants (diagnosed at less than 1 year of age) with acute lymphoblastic leukemia (ALL) and in young to middle-aged adults with acute myeloid leukemia (AML) (1, 2). The outcome of KMT2A-rearranged (KMT2A-r) leukemia is characteristically poor, especially in infants. The 5-year event-free survival (EFS) for infant KMT2A-r ALL remains below 50% despite the implementation of risk stratification and the use of intensified chemotherapy treatment protocols and stem cell transplantation in high-risk infants (3). Moreover, the established treatment protocols have detrimental long-term health effects. This is particularly relevant in the setting of infant and childhood KMT2A-r leukemia, as these young patients receive high doses of chemotherapeutic agents for extended periods of time, often resulting in developmental delay and decreased quality of life decades later (4). Given that the number of childhood leukemia survivors is on the rise, the detrimental consequences of conventional treatments on long-term health are becoming ever more evident. The search for more potent and safer treatment strategies for KMT2A-r leukemia thus remains an area of pressing unmet clinical need.

We previously found that a novel, non-genotoxic drug, curaxin CBL0137, currently in clinical trials for adult and pediatric malignancies (NCT03727789, NCT01905228, NCT04870944), inhibited leukemia progression and extended survival in KMT2A-r leukemia models, including infant KMT2A-r ALL (5). CBL0137 exerts its anti-cancer effect through intercalating into DNA and inducing genome-wide nucleosome destabilization that activates a cascade of anti-cancer effects, including trapping the histone chaperone Facilitates Chromatin Transcription (FACT) on chromatin, inhibition of DNA damage repair, p53 activation, NFκB suppression, and induction of TRAIN (Transcription of Repeats Activates Interferon) (5–7).

Effective therapeutic management of high-risk cancers requires innovative combination strategies. While we showed that CBL0137 impressively potentiates standard of care chemotherapies in models of KMT2A-r leukemia (5), the identification of a potent and well-tolerated combination of CBL0137 with a more targeted therapeutic approach is of high clinical relevance. Based on previous studies highlighting the promising preclinical efficacy of histone deacetylase (HDAC) inhibitors against KMT2A-r leukemia (8, 9), we here investigated the preclinical efficacy of a novel combination of targeted drugs, CBL0137 and the HDAC inhibitor panobinostat, in KMT2A-r leukemia models.

## Materials and Methods

### Chemicals and Reagents

CBL0137 was provided by Incuron, Inc. Panobinostat and entinostat (MS-275) were purchased from Sapphire Bioscience Pty Ltd. (Redfern, New South Wales, Australia).

### Cell Lines, Cell Culture and Cell-Based Assays

All cell lines used in this study were mycoplasma-free and have been authenticated using STR profiling in the past 3 years. Cell culturing and assays measuring apoptosis and cell cycle were performed as previously described (10, 11). In apoptosis assays, cells were stained with annexin V and 7-Aminoactinomycin D (7-AAD) according to supplier specifications (BD Biosciences, North Ryde, New South Wales, Australia). In synergy viability assays, cells were treated with increasing doses of drugs in a 6x6 combination matrix format. Cell viability was measured by resazurin reduction-based assays after a 5-day treatment. Synergy was scored according to the Bliss Independence model and visualized by Combenefit as previously described (12).

### Soft Agar Colony Formation Assays

Six-well plates were coated with a layer of RPMI medium containing 10% FBS and 0.5% agar (agarose, Lonza). Cells were seeded at a density of 800 cells/well in RPMI containing 10% fetal bovine serum, 0.33% agar in the presence of vehicle, CBL0137, panobinostat or the combination. After 14 days, colonies were stained with MTT and counted as previously described (13). In synergy colony assays, cells were treated at incremental doses of CBL0137 or panobinostat as single agent or at a fixed 50:1 ratio for the combination. The occurrence of synergy was assessed based on the Bliss Independence model as previously described (14). Bliss prediction curves indicate the predicted percentage of colonies of treated cells relative to controls when the combination of compounds works additively together. Synergy is visualized as the presence of a lower percentage of colonies upon combination of two compounds compared to the colony percentage predicted based on the presence of an additive effect of the compounds (i.e., the dose-response curve of the combination runs below the Bliss prediction curve).

### Western Blotting

Western blotting on lysates from cells and mouse splenocytes was performed as previously described (12). The following antibodies were used: anti-P53 (554293, BD Biosciences), anti-γH2AX (97185, Cell Signaling Technology, New England Biolabs, Notting Hill, Victoria, Australia), anti-IFIT3 (ABF1048, Millipore, Bayswater, Victoria, Australia) anti-H3Ac (06-599, Millipore), anti-H3K27Ac (4353S, Cell Signaling Technology), anti-Histone H3 (total, 9715, Cell Signaling Technology), anti-H3K4Me3 (9751, Cell Signaling Technology), anti-actin (A2066, Sigma, North Ryde, New South Wales, Australia), anti-GAPDH (2118, Cell Signaling Technology).

### IP-10 AlphaLISA

IP-10 concentrations in mouse serum samples were quantified using the mIP-10/CXCL10 AlphaLISA immunoassay kit (PerkinElmer, Waverley, Victoria, Australia) according to manufacturers’ instructions with minor modifications. Using a 1536-wells Alphaplate (PerkinElmer), 1 µl AlphaLISA buffer was added to each well, followed by 0.25 µl diluted serum, then 0.25 µl of 10x acceptor/biotin mix. After 1 hour, 1 µl of 2.5x donor beads was added, incubated for another hour before reading the plate on the PerkinElmer Envision. All sample and bead additions were done using the Echo acoustic dispenser (Beckman Coulter, Lane Cove, New South Wales, Australia). The mIP-10 standard curve was made up in fetal bovine serum (FBS) and all test samples were diluted 4-fold in FBS due to signal inhibition by undiluted sample matrix. Spike recovery experiments showed good recovery (>80%) after >4-fold sample dilution. The assay lower detection limit was determined to be 100 pg/ml.

### RNA Isolation and qRT-PCR

RNA isolation and qRT-PCR were performed as described (11, 15).

### Histone Isolation and Dot Blotting

Histones were purified from splenocytes isolated from treated patient-derived xenograft mice with the EpiQuik Total Histone Extraction Kit (Epigentek, Sapphire Bioscience Pty. Ltd.) according to the manufacturers’ instructions. 0.375 µg histone prep (in 1.5 µl total volume) was spotted onto a nitrocellulose membrane. After drying of the membrane, the staining protocol for Western blotting was performed.

### Animal Experiments

All animal experiments were approved by the University of New South Wales Animal Care and Ethics Committee according to the Animal Research Act 1985, and the Australian Code of Practice for Care and Use of Animals for Scientific Purposes (2013). Efficacy studies with CBL0137 and panobinostat in the MLL-AF9;NRas^G12D^ AML mouse model and the MLL-6 KMT2A-r ALL patient-derived xenograft (PDX) model were performed in C57BL/6 and NOD/SCID mice respectively, as previously described (5, 12, 16, 17). Individual mouse event-free survival (EFS) was calculated as the days from treatment initiation until mice reached a total score of 3 in body condition scoring (based on coat condition, activity and body weight) together with bioluminescent evidence of engraftment in the MLL-AF9 (*KMT2A:MLLT3)*;NRas^G12D^ AML model or until the %huCD45^+^ cells reached 25% in the MLL-6 PDX model. Applied drug doses were determined based on maximally tolerated dose studies and previously performed efficacy studies. In the MLL-AF9;NRas^G12D^ AML mouse model, 60 mg/kg CBL0137 was administered *via* oral gavage twice a week for two weeks. Panobinostat (7.5 mg/kg) or vehicle (5% dextrose) was administered intraperitoneally five days/week for two weeks. In the KMT2A-r ALL PDX model, CBL0137 (45 mg/kg in 5% dextrose) was administered intravenously twice a week for three weeks. Panobinostat (5 mg/kg) was administered intraperitoneally five days/week for two weeks. For combination studies, panobinostat and CBL0137 were administered according to their individual regimens. To evaluate interactions between drugs *in vivo*, Therapeutic Enhancement was concluded if the EFS of mice treated with the drug combination was significantly greater (P < 0.01) than the EFS induced by both single agents as determined by Gehan-Wilcoxon (12, 18).

### Statistical Analysis

GraphPad Prism Version 8 was used for all statistical analyses. EFS curves were compared by Gehan-Wilcoxon analysis. Comparisons of variables between groups were performed as indicated in individual figure legends. P values <0.05 were considered significant.

## Results

### The Combination of CBL0137 and Panobinostat Delays Leukemia Progression in an Aggressive Syngeneic Mouse Model of MLL-AF9 AML

**Figure 1:**
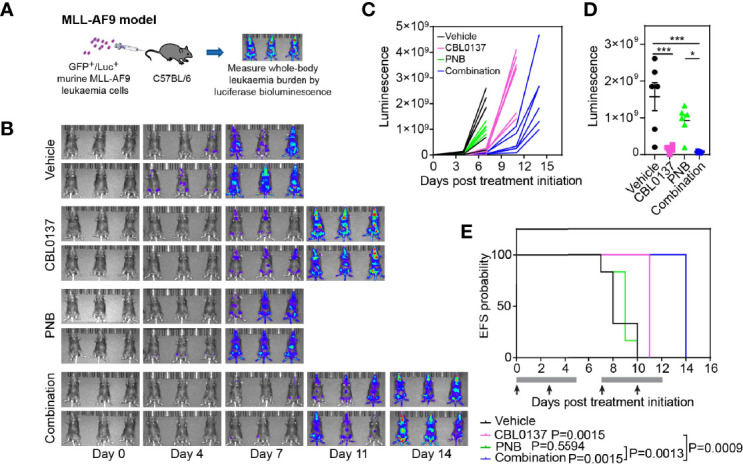
The combination of CBL0137 and panobinostat (PNB) limits KMT2A-r acute myeloid leukemia progression. **(A)** Schematic of the MLL-AF9/NRas^G12D^ AML mouse model. **(B)** Whole body bioluminescence imaging at different time points after MLL-AF9/NRas^G12D^ leukemia cell inoculation. **(C)** Quantification of whole-body bioluminescent imaging in individual mice. **(D)** Comparison of whole-body bioluminescent signal on day 8 after inoculation. Each dot corresponds to the signal in one mouse. The significance of the difference between group mean signals was determined by ANOVA followed by Tukey’s multiple comparison test. **(E)** Kaplan-Meier survival plot. The solid bar below the survival graphs represents treatment duration for PNB and arrows indicate CBL0137 administration. The Gehan-Wilcoxon test was used to compare survival between treatment groups. P-values comparing survival of control and treatment groups are denoted directly next to the legend. Statistical data displayed at the top of a bar in the graphs correspond to comparisons with controls. Asterisks represent significance levels of P values: *P < 0.05, ***P < 0.001.

We previously showed that curaxin CBL0137 inhibits leukemia progression in KMT2A-r leukemia models (5). To investigate whether the anti-leukemic effect of the drug is potentiated when combined with HDAC targeting, we tested the combination of CBL0137 and the HDAC inhibitor panobinostat in a highly aggressive, transplantable mouse model of MLL-AF9 (*KMT2A:MLLT3)* leukemia. Mouse fetal liver cells were transduced with MSCV-MLL-AF9-IRES-VENUS and MSCV-luciferase-IRES-NRas^G12D^ expression constructs (MLL-AF9;NRas^G12D^) as previously described (16, 17). These cells were introduced into primary recipient mice to induce leukemogenesis and then inoculated into secondary recipients for subsequent studies (**Figure 1A**) (16, 17). Leukemia engraftment and progression in control mice progressed rapidly as determined by whole-body bioluminescence imaging (**Figure 1B**). Treatment with CBL0137 and panobinostat, either alone or in combination, was started on day 4 after inoculation when all mice presented with evidence of bone marrow engraftment based on bioluminescence signals.

CBL0137 significantly decreased leukemia burden compared to vehicle-treated mice at any given time point (**Figures 1B–D** and **Supplementary Figure 1A**) and significantly extended event free survival (EFS, CBL0137 vs control: P=0.0015, **Figure 1E** and **Table 1**). In line with the potential of CBL0137 to induce an interferon response (15, 19, 20), the serum of mice treated with CBL0137 contained augmented levels of the chemokine Interferon γ-induced protein 10 (IP-10, CXCL10) (**Supplementary Figure 2A**) and splenocytes expressed higher levels of IFIT3 (**Supplementary Figure 2B**), demonstrating target engagement. Panobinostat alone, despite inducing accumulation of DNA damage in splenocytes of treated mice as demonstrated by increased levels of γH2AX (**Supplementary Figure 2B**), did not significantly change average leukemia burden or survival (**Figures 1B–E**). The combination of CBL0137 and panobinostat was well tolerated (**Supplementary Figure 1A**) and suppressed leukemia progression to the greatest extent, reducing whole body leukemia burden on average by approximately 30-fold compared to control mice one week after treatment initiation (**Figure 1D**). The combination provided a further therapeutic enhancement with an EFS extension of 75% compared to control mice and significantly extended EFS compared to cohorts treated with either single agent (combo vs panobinostat: P=0.0013; combo vs CBL0137: P=0.0009, **Figure 1E** and **Table 1**). Thus, the combination of CBL0137 and panobinostat exerts markedly enhanced inhibitory effects against this aggressive KMT2A-r AML model compared with either drug alone.

**Table 1:** Summary of *in vivo* efficacy of CBL0137 combined with panobinostat.

	Treatment Group	Animal Counts/Status					EFS Evaluation						Response Evaluation^11^				
		N^1^	Nd^2^	Nx^3^	Na^4^	Nev^5^	KMmed^6^	EFS_T - C_^7^	EFS_T/C_^8^	P-value^9^	P-value^10^	PD	SD	PR	CR	Overall group response
MLL-AF9/NRas^G12D^	Vehicle	6	0	0	6	6	8.0									
	CBL0137	6	0	0	6	6	11.0	3.0	1.38	0.0015	0.0009					
	Panobinostat	6	0	0	6	6	9.0	1.0	1.13	0.5594	0.0013					
	CBL0137 + Panobinostat	6	0	0	6	6	14.0	6.0	1.75	0.0015						
															
MLL-6	Vehicle	8	0	0	8	8	10.1					8	0	0	0	PD
PDX	CBL0137	8	0	0	8	8	23.3	13.2	2.31	0.0002	0.0084	7	0	0	1	PD
	Panobinostat	8	0	0	8	8	18.7	8.6	1.86	0.0055	0.0011	8	0	0	0	PD
	CBL0137 + Panobinostat	8	2	0	6	6	32.8	22.7	3.25	0.0011		1	0	4	1	PR

^1^N = total number of mice entering experiment.

^2^Nd = number of mice experiencing treatment-related death.

^3^Nx = number of additional mice excluded from analysis.

^4^Na = number of mice in analysis.

^5^Nev = number of events, defined as hCD45+ cells ≥ 25%.

^6^KM med = Kaplan-Meier estimate of median time-to-event (days).

^7^EFS _T – C_ = Leukemia Growth Delay (LGD) = difference in median time-to-event (days) between treated (T) and control (C) groups.

^8^EFS _T/ C_ = relative difference in median time-to-event (days) between T and C groups.

^9^P-value comparing EFS (treatment group) to EFS (vehicle-treated group), computed using Gehan-Wilcoxon test.

^10^P-value comparing EFS (combination treatment group) to EFS (single agent-treated group), computed using Gehan-Wilcoxon test.

^11^PD, progressive disease: if the %huCD45+ never dropped below 1% and mice reached event before the end of the study period (42 days post-treatment initiation);

SD, stable disease: if the %huCD45+ never dropped below 1% but event was not reached before the end of the study period;

PR, partial response: if the %huCD45+ dropped below 1% at any one time point regardless of whether an event is reached before the end of the study;

CR, complete response: if the %huCD45+ dropped below 1% for two consecutive weeks.

### The Combination of CBL0137 and HDAC Inhibition Decreases KMT2A-r Leukemia Cell Viability

**Figure 2:**
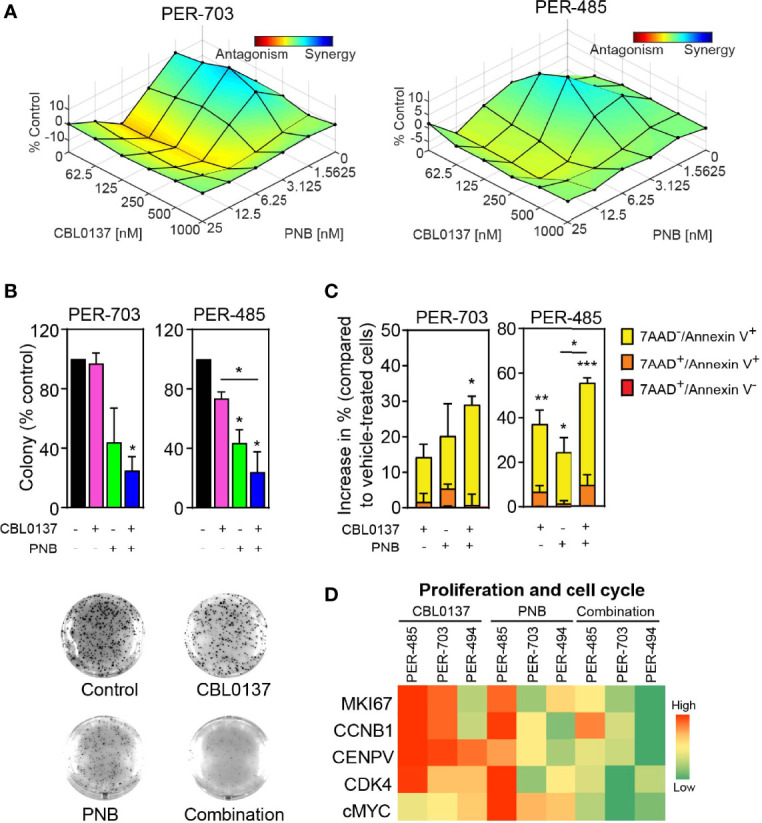
The combination of CBL0137 and panobinostat (PNB) kills KMT2A-r leukemia cells. **(A)** Synergy distribution according to Bliss and visualized by Combenefit. Cells were treated with increasing doses of CBL0137 combined with panobinostat in a 6 × 6 matrix format and cell viability was measured by resazurin reduction-based assays after 5 days (n=2). **(B)** Soft-agar colony assays with infant KMT2A-r leukemia cells treated with CBL0137 (PER-703: 0.2 µM; PER-485: 0.3 µM) and/or PNB (PER-703: 4 nM; PER-485: 6 nM) for 10 to 14 days. Bar graphs represent the mean % colonies relative to vehicle-treated cells +/- SEM of two independent biological replicates. Representative images of PER-703 treatment wells are shown under the graph. **(C)** Mean percentage increase in annexin V^+^ PER-485 and PER-703 cells (includes annexin V^+^/7AAD^-^ and annexin V^+^/7AAD^+^ cells) relative to vehicle-treated cells after treatment with CBL0137 (0.3 µM), PNB (10 nM) or the combination for 24 hours. Graphs depict mean ± SE of at least three independent experiments. Statistical analyses were performed for the sum of all annexin V^+^ cells (7AAD^-^ and 7AAD^+^). **(D)** Heatmap of mRNA expression of genes in treated KMT2A-r leukemia cells relative to vehicle-treated cells as determined by qRT-PCR. Statistical data displayed at the top of a bar in the graphs correspond to comparisons with control cells. Statistical significance was determined by one-way ANOVA. Asterisks represent significance levels of P values: *P < 0.05; **P < 0.01; ***P < 0.001.

We next investigated whether the observed anti-leukemic effects of the CBL0137/panobinostat drug combination could be confirmed in human-derived KMT2A-r leukemia cells. We firstly assessed the impact of CBL0137 and panobinostat, as single agents and in combination, on the viability of low-passage leukemia cell lines derived from infants with poor outcome KMT2A-r ALL in formal synergy assays. Infant KMT2A-r leukemia cells (PER-703, PER-485, PER-494) were exposed to CBL0137 or panobinostat either alone or in combination in a 6x6 combination matrix and the effect of the drugs on cell viability was assessed in resazurin-reduction based viability assays. Synergy analysis was performed on the viability data according to the Bliss Independence model and showed the occurrence of mainly additive effects between the two drugs (**Figure 2A** and **Supplementary Figures 3**, **4**) (21). The additive nature of the CBL0137/panobinostat combination was subsequently confirmed in longer term colony formation assays (**Supplementary Figure 5**). While no convincing synergy was observed between CBL0137 and panobinostat in KMT2A-r leukemia cells *in vitro*, for certain drug concentration combinations, significantly decreased colony numbers were observed after combination treatment compared to vehicle-treated cells (**Figure 2B**). The observed additive inhibitory effect of the combination of CBL0137 and panobinostat on the viability of KMT2A-r leukemia cells was not specific to KMT2A-translocated leukemias and was also observed for KMT2A-wildtype leukemias (**Supplementary Figure 6**).

Given that curaxin CBL0137 inhibits KMT2A-r leukemia cell growth by rapidly inducing apoptosis (5), we next determined whether HDAC inhibition boosted CBL0137-induced apoptosis by measuring the level of annexin V^+^ cells. Treatment of PER-485 and PER-703 cells with the CBL0137 and panobinostat combination significantly increased the percentage of annexin V^+^ cells within 24 hours of treatment compared to vehicle treatment (**Figure 2C**), indicative of rapid induction of apoptosis. Only minor effects were observed on cell cycle progression of cells treated with the combination (**Supplementary Figure 7**). The observed effects of the combination on KMT2A-r leukemia cell survival were associated with downregulated expression of genes involved in proliferation and cell cycle progression including *MKI67*, *CCNB1*, *CENPV*, *CDK4* and *cMYC* in infant KMT2A-r leukemia cells PER-485, PER-703 and PER-494 (**Figure 2D**).

Similarly, a significantly increased level of apoptotic cells was observed when infant KMT2A-r leukemia cells were treated with CBL0137 in combination with another HDAC inhibitor, entinostat (**Supplementary Figure 8**), confirming that the enhanced anti-leukemic effect provided above by panobinostat in combination treatment involves its inhibitory action on deacetylation.

KMT2A-r leukemia is characterized by chromatin remodeling, epigenetic modifications and changes in histone marks (22, 23). As both CBL0137 and panobinostat impact chromatin structure, we investigated whether the additive effects of the drug combination were mediated by changes in histone acetylation levels or in histone marks associated with *KMT2A* translocations. We assessed the effect of *in vitro* treatment with the single agents and drug combination on histone H3 acetylation levels (total H3Ac and H3K27Ac), predicted to be impacted by panobinostat, and levels of trimethylated H3K4 (H3K4me3), a histone mark implicated in the pathobiology of *KMT2A* rearrangements (22, 24). While panobinostat, as expected, increased levels of acetylated total histone H3 and H3K27, the combination of CBL0137 and panobinostat did not further enhance this effect (**Supplementary Figure 9**). Similarly, while panobinostat treatment boosted the levels of tri-methylated H3K4 (H3K4me3) as previously described (24), the addition of CBL0137 did not further increase H3K4me3 levels. In line with the absence of an enhanced effect by the CBL0137/panobinostat drug combination on histone marks in KMT2A-r leukemia cells, we observed that the drug combination did not change expression levels of *HOXA9* and *MEIS1* genes, well-described leukemogenic target genes of KMT2A-r fusion proteins and upregulated in many KMT2A-r leukemias downstream of epigenetic modifications driven by *KMT2A* translocations (**Supplementary Figure 10**) (22).

### The Combination of CBL0137 and Panobinostat Exhibits Potent Anti-Leukemic Activity in an Infant KMT2A-r ALL Patient-Derived Xenograft (PDX) Model

**Figure 3:**
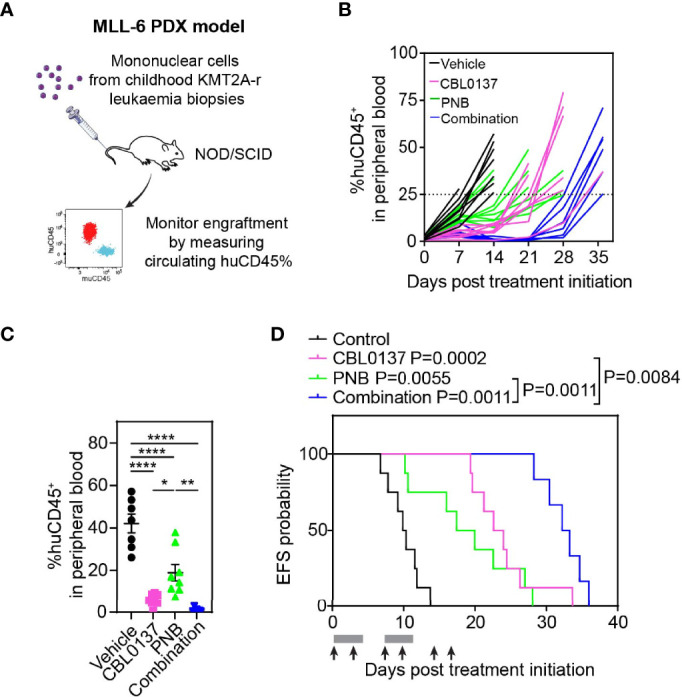
The combination of CBL0137 and panobinostat (PNB) limits infant KMT2A-r ALL PDX progression. **(A)** Schematic of the infant KMT2A-r ALL PDX model. **(B)** Leukemia cell levels as measured by enumeration of the % huCD45^+^ cells in the peripheral blood of individual mice over time. **(C)** Comparison of % huCD45^+^ cells in blood of treated mice on day 14 post treatment initiation. Each dot corresponds to one mouse. Statistical significance of differences in mean engraftment between groups was determined by ANOVA followed by Tukey’s multiple comparison test. **(D)** Kaplan-Meier survival plot with endpoint defined as %huCD45^+^ cells in the peripheral blood reaching 25% or higher. The solid bar below the survival graphs represents treatment duration for PNB and arrows indicate CBL0137 administration. The Gehan-Wilcoxon test was used to compare survival between treatment groups. P-values comparing survival of control and treatment groups are denoted directly next to the legend. Asterisks represent significance levels of P values: *P < 0.05, **P < 0.01; ****P < 0.0001.

We subsequently examined the efficacy of the CBL0137 and panobinostat combination in a highly aggressive human PDX model of infant KMT2A-r ALL (MLL-6), which harbors a *KMT2A:MLLT1* rearrangement (25, 26). PDX cells were inoculated intravenously into NOD/SCID mice, and leukemia engraftment and progression were assessed weekly by determining the percentage of circulating human CD45^+^ (huCD45^+^) leukemia cells as a proportion of total CD45^+^ cells (**Figure 3A**). Treatment was started when the median %huCD45^+^ cells in the blood exceeded 1% and drug efficacy was evaluated based on the circulating levels of huCD45^+^ cells as previously described (12, 27).

CBL0137 and panobinostat were administered according to administration regimens that were previously shown to enable target engagement (5, 15). CBL0137 induced the activation of the P53 pathway as previously described and treatment with panobinostat increased levels of acetylated histone H3 and H3K27 in leukemic blasts in mouse spleens, confirming effective inhibition of histone deacetylase activity by the drug (**Supplementary Figure 11**).

Leukemia progressed quickly in control mice with median EFS of just 10 days (**Figure 3B**; **Supplementary Figure 12A**; **Table 1**). Treatment was well tolerated with no significant weight loss in the majority of mice (**Supplementary Figure 12B**; **Table 1**). While leukemia engraftment was delayed in mice treated with CBL0137 or panobinostat as a single agent, the combination of both drugs was able to reduce circulating levels of leukemic blasts below 1% throughout a substantial proportion of the treatment period in the majority of mice (**Figures 3B, C**). However, in line with our *in vitro* studies, this enhanced effect by the combination was not associated with increased levels of acetylated H3 or H3K27, or changes in levels of *KMT2A* translocation associated histone marks H3K4me3 or H3K79me2 compared to the single agent treatment (**Supplementary Figure 11B**) (22).

CBL0137 and panobinostat as monotherapies induced EFS extensions of 131% and 86% compared to control mice, respectively, and significant differences were observed between the median EFS of the treated cohorts versus control (CBL0137 vs control: P=0.0002, panobinostat vs control: P=0.0055, **Figure 3D**; **Table 1**). The combination of CBL0137 and panobinostat provided further therapeutic enhancement with a significantly extended EFS (22.7 days or 225% compared to control animals) compared to cohorts treated with either single agent (P=0.0011 combo vs panobinostat; P=0.0084 combo vs CBL0137, **Figure 3D**; **Table 1**). In fact, the fold-change in EFS between the combination treatment and control groups (EFS_treated_/EFS_control_ = 3.25, **Table 1**) is comparable to that produced by an ALL induction therapy-based regimen, VXL (vincristine, dexamethasone, and *L*-asparaginase) (EFS_treated_/EFS_control_ = 3.18) or the combination of CBL0137 and cyclophosphamide (EFS_treated_/EFS_control_ = 3.35), which we have tested previously in the same MLL-6 PDX model (5).

In addition, we evaluated the therapeutic response to the drugs by using an objective response measure that is based on strict criteria modelled after the assessment of patient leukemia cell numbers as performed in the clinic (27). A drug is defined as achieving an objective response in a mouse when treatment reduces circulating leukemia levels below 1% at any point during the study (27). Thus, the combination of CBL0137 and panobinostat achieved an objective response (partial response) in this aggressive infant KMT2A-r ALL PDX model while cohorts treated with the same doses of each agent individually displayed progressive disease, supporting the clinical potential of this drug combination (**Table 1**).

## Discussion

Based on the urgent need to identify better and safer treatment options for leukemias driven by a rearrangement of the *KMT2A* gene, we report on a novel, clinically applicable combination of anti-cancer drugs, the curaxin CBL0137 and the HDAC inhibitor panobinostat, that displays significant efficacy in aggressive animal models of KMT2A-r leukemia. In contrast to several other therapies for KMT2A-r leukemias that are focused on targeting the disease-associated aberrant epigenome, this drug combination rapidly induces apoptotic cell death, within one to two days of treatment *in vitro*.

Both CBL0137 and panobinostat decondense chromatin respectively by destabilising nucleosomes and by increasing histone acetylation levels. These ‘chromatin damaging’ effects are thought to selectively impact cancer cells as these cells often exhibit, and are dependent on, altered chromatin morphology (28). Chromatin decondensation provokes a host of downstream anti-cancer processes. The observed inhibitory effects of chromatin damaging drugs on cancer cells can result from modulation of different downstream anti-cancer mechanisms, depending on the specific wiring of the cancer type under investigation. This is showcased by our previous findings on the effectiveness of the combination of CBL0137 and panobinostat in models of other high-risk pediatric cancers including neuroblastoma and diffuse intrinsic pontine glioma (DIPG) (15, 29). The CBL0137/panobinostat combination induced enhanced interferon signaling which increased the combination efficacy in immunocompetent neuroblastoma models, while the restoration of histone H3 acetylation and trimethylation was deemed to be critical for the boosted therapeutic effects of the combination in preclinical models of DIPG harboring mutant histone H3 (15, 29).

To our knowledge, this is the first report on the efficacy of the CBL0137/panobinostat combination approach to treat pediatric hematological malignancies. As KMT2A-r leukemia is driven by aberrant, oncogenic KMT2A fusion proteins that activate a hematopoietic stem cell-like transcriptional program by modifying the cell’s epigenome and chromatin structure (30), this leukemia subtype would in principle make an excellent target for drugs that affect chromatin organization. In particular, infants with KMT2A-r ALL would be ideal candidates given that the disease is primarily driven by the leukemogenic KMT2A fusion gene signature with the near absence of other driver mutations (31). However, the CBL0137/panobinostat combination did not induce significant changes in the levels of acetylation or methylation of specific histones associated with KMT2A-r leukemia or in the expression levels of leukemogenic *KMT2A* target genes such as *HOXA9* and *MEIS1* before induction of apoptosis. This indicates that the observed therapeutic enhancement by the drug combination is not mediated through a concerted effect of the drugs on histone marks. However, we did observe attenuation of the expression of several genes involved in cell proliferation and cell cycle, including *cMYC* in KMT2A-r leukemia cells treated with the drug combination. This is in agreement with other studies reporting that CBL0137 as well as panobinostat attenuate *MYC* oncogene expression in cancer cells (32, 33).

The observed cooperative effects of the CBL0137 and panobinostat combination appeared less pronounced *in vitro* than in the *in vivo* setting where the combination showed significant delays in leukemia progression compared to single agents in two highly aggressive KMT2A-r leukemia models. These results suggest that additional changes in the tumor microenvironment induced by the drugs may further contribute to the observed therapeutic enhancement. On the other hand, it is becoming increasingly clear that the true mechanistic basis for the success of multidrug combinations in animals and humans *in vivo* remains largely obscure (34, 35). Recent studies by Palmer *et al.* indicate that independent drug actions and variability between animals/patients are sufficient to explain the superiority of many approved drug combinations and that drug synergy or additivity might not come into play (34, 35). This implies that caution is warranted in interpreting the significance of putative mechanisms of synergy or enhancement identified in *in vitro* and *in vivo* experimental models.

Nevertheless, while the exact underlying mechanisms of therapeutic enhancement for this combination treatment in KMT2A-r leukemia remain difficult to delineate, the observed enhancement of treatment response upon combining these drugs in our animal models showcases the potential clinical significance of the drug combination and provides impetus for further preclinical and clinical evaluation. It will thereby be of interest to investigate how this drug combination can be incorporated into current treatment regimens. Despite our finding that the CBL0137/panobinostat drug combination significantly extends survival, all leukemia-engrafted mice still succumbed to disease. This suggests that the drug combination may have most value as an add-on therapy, administered simultaneously with standard of care agents or added into the treatment scheme sequentially after standard of care, rather than as a standalone treatment.

In conclusion, this study proposes the combination of CBL0137 and panobinostat as a candidate therapeutic approach for KMT2A-r leukemia that warrants further investigation.

## Data Availability Statement

The raw data supporting the conclusions of this article will be made available by the authors, without undue reservation.

## Ethics Statement

The animal study was reviewed and approved by the University of New South Wales Animal Care and Ethics Committee.

## Author Contributions

MK, LX, AB, ER, AK, AE-A, JS, and KS conducted the experiments. MK, LX, ER, and KS analyzed the data. JS and BC provided guidance on cell cycle experiments. LC and RK provided guidance and access to the cell lines used in the study. JR and GA executed and analyzed the AlphaLISA for IP-10. AN, SB, and RJ provided guidance on experiments with the MLL-AF9/NRas^G12D^ model and access to cells used for transplantation. RL provided guidance and access to PDX cells. LX, MH, MN, MJH, and KS conceived the project and designed the experiments. AG and KG provided support with study design. LX and KS wrote the manuscript under the guidance of MH, MJH, and MN who critically reviewed the manuscript. All authors contributed to the article and approved the submitted version.

## Funding

LX has received grants from Cancer Institute NSW (ECF171127), Tour de Cure and Neuroblastoma Australia. KS is supported by funding from the Kids Cancer Alliance (KCA). KS, MJH, MH, and MN are supported by a grant awarded through the Priority-driven Collaborative Cancer Research Scheme and co-funded by Cancer Australia and The Kids’ Cancer Project (APP1164865) and philanthropy from Tenix Foundation. SB is supported by a Cancer Council Victoria (CCV) Postdoctoral Fellowship. The laboratory of RJ was supported by the Cancer Council of Victoria, National Health and Medical Research Council of Australia (NHMRC) and The Kids’ Cancer Project, and the Peter MacCallum Foundation and Australian Cancer Research Foundation provide generous support for equipment and core facilities. RL is supported by National Health and Medical Research Council of Australia (NHMRC) Fellowships APP1059804 and APP1157871. MJH and MH are recipients of a grant from Anthony Rothe Memorial Trust for this work. MH and MN are supported by grants from NHMRC (APP1132608 and APP1085411), Cancer Institute NSW (14/TPG/1-13) and Cancer Council NSW (PG 16-01). LC and RK are supported by the Children’s Leukemia and Cancer Research Foundation.

## Conflict of Interest

KG and AG are co-authors of patent WO2010/042445 “Carbazol compounds and the therapeutic use of the compounds”. KG was a recipient of research grants and consulting payments from Incuron, Inc. The laboratory of RJ receives research support from F. Hoffmann-La Roche Ltd., Astra Zeneca, BMS, and MecRx. RJ is a scientific consultant and shareholder in MecRx.

The remaining authors declare that the research was conducted in the absence of any commercial or financial relationships that could be construed as a potential conflict of interest.

## Publisher’s Note

All claims expressed in this article are solely those of the authors and do not necessarily represent those of their affiliated organizations, or those of the publisher, the editors and the reviewers. Any product that may be evaluated in this article, or claim that may be made by its manufacturer, is not guaranteed or endorsed by the publisher.
