# Supplementary material for: The Combination of Curaxin CBL0137 and Histone Deacetylase Inhibitor Panobinostat Delays KMT2A-Rearranged Leukemia Progression
Source: Front Oncol. 2022 May 23;12:863329. doi: 10.3389/fonc.2022.863329 (PMC9168530; doi:10.3389/fonc.2022.863329)
Supplement: Supplementary file 1 [file Presentation_1.pdf]

Supplementary Material

Supplementary Figures

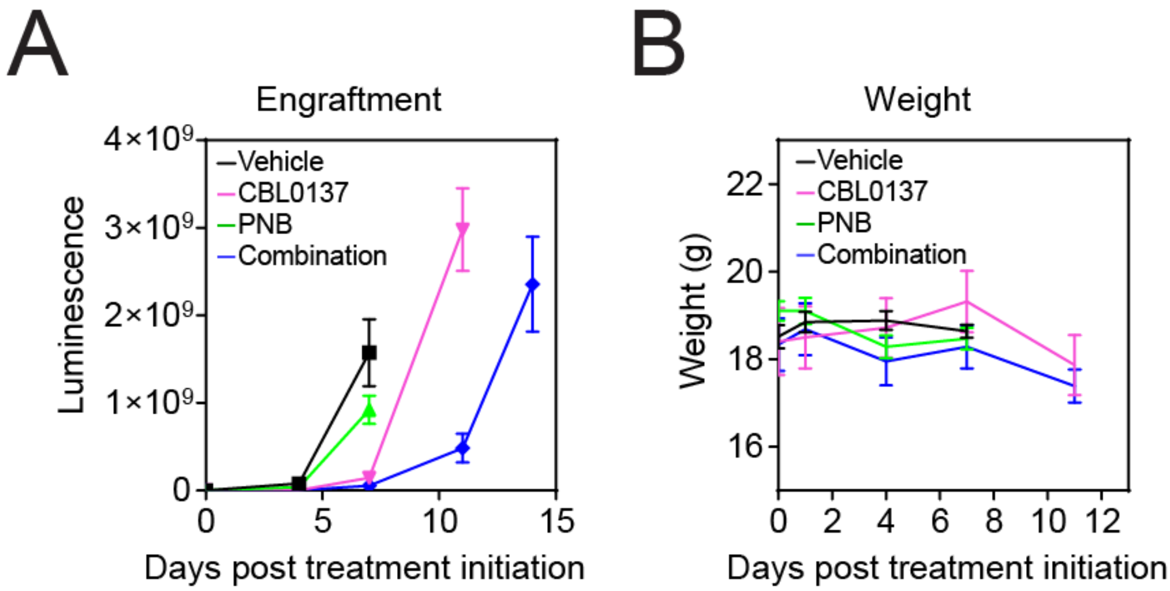

Supplementary Figure 1. The combination of CBL0137 and panobinostat (PNB) limits disease progression in a mouse KMT2A-r acute myeloid leukemia model. **(A)** Quantification of whole-body bioluminescent signal in individual mice. Graph shows the mean signal per group of the individual mice shown in Fig. 1C (n=6 mice per group) ± SE. **(B)** Evolution of mean weight per group ± SE.

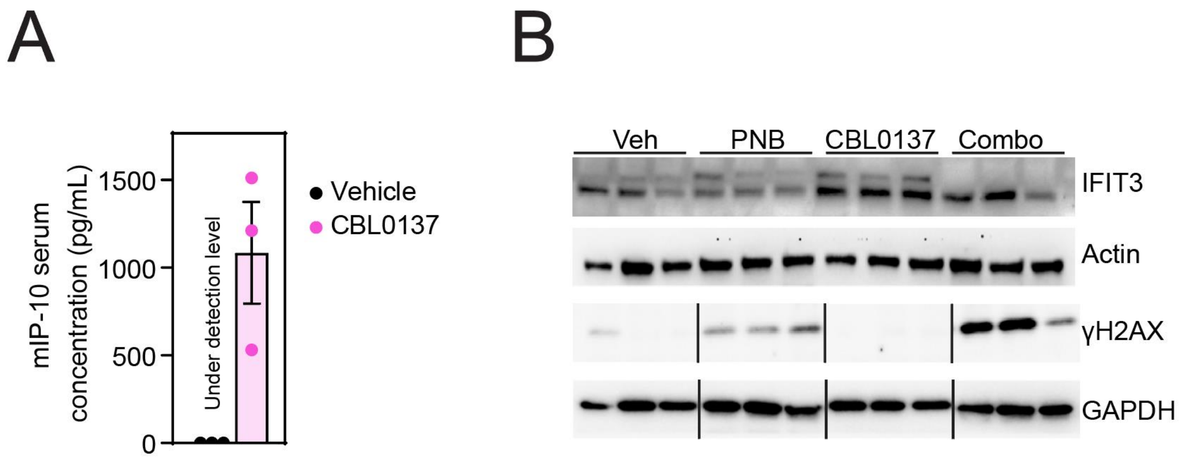

Supplementary Figure 2. (A) Serum IP-10 concentration in MLL-AF9/NRas^{G12D} engrafted C57BL/6 mice treated with either vehicle or CBL0137 as quantified using AlphaLISA immunoassays. Graph shows the mean ± SE (n=3 mice per group). **(B)** Western blots showing expression of IFIT3 and γH2AX in splenocytes harvested from mice treated with either vehicle, Panobinostat (PNB), CBL0137, or the combination (n=3 mice per group).

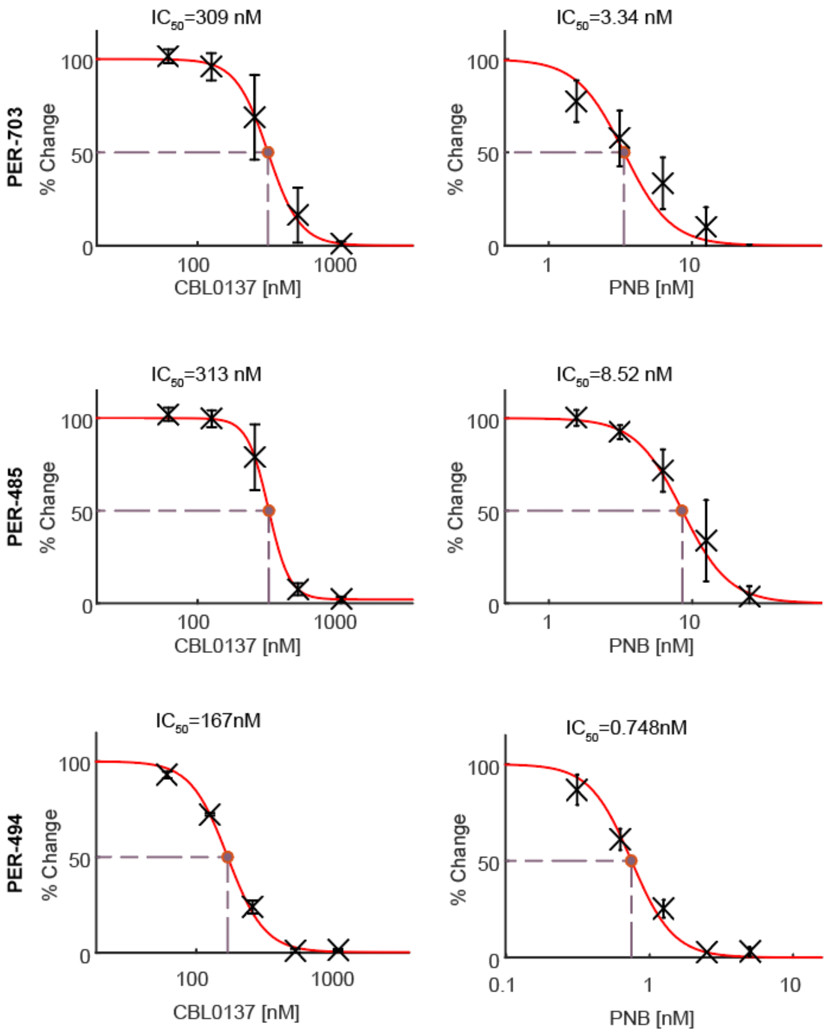

Supplementary Figure 3. Single agent viability curves of KMT2A-r leukemia cells after a 5-day treatment with CBL0137 or panobinostat (PNB), as assessed in 6x6 matrix synergy viability assays (corresponding to the synergy data displayed in Figure 2A). Graphs show the mean percentage viability (relative to vehicle-treated cells) of three independent replicates ± SE for PER-485 and PER-703 cells and two independent replicates ± SE for PER-494 cells.

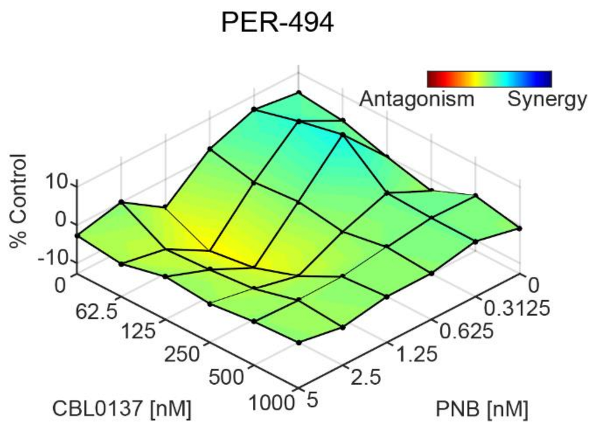

Supplementary Figure 4. Synergy analysis of PER-494 cells treated with CBL0137, panobinostat (PNB) or the CBL0137/PNB combination. Cells were incubated with increasing doses of CBL0137, PNB or the combination in a 6×6 matrix format and cell viability was measured by resazurin reduction-based assays after 5 days (n=2). Synergy was assessed according to Bliss and visualized by Combenefit.

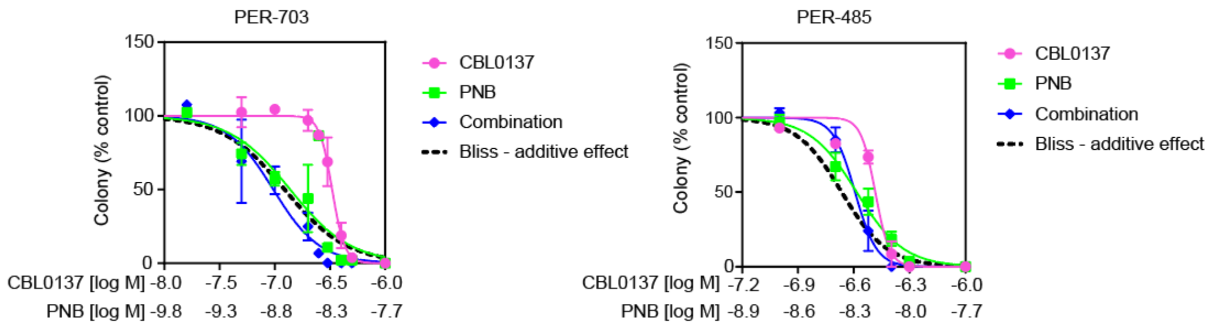

Supplementary Figure 5. Dose response curves displaying the percentage of colonies relative to vehicle-treated cells when cells are exposed to CBL0137, panobinostat (PNB) or the combination in soft agar colony assays. Graphs depict mean ± SE of at least two independent experiments. Drug synergy was calculated by applying the Bliss additivity model. Dotted lines indicate predicted effect if compounds are additive and combination curves (blue) below the dotted Bliss line indicate the occurrence of synergy between the tested drugs.

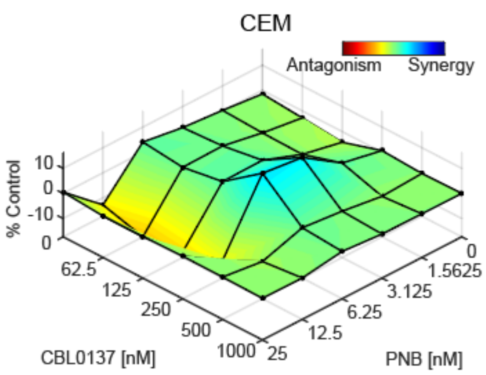

Supplementary Figure 6. Synergy analysis of KMT2A-wildtype CEM and REH leukaemia cells treated with CBL0137, panobinostat (PNB) or the CBL0137/PNB combination. Cells were incubated with increasing doses of CBL0137, PNB or the combination in a 6×6 matrix format and cell viability was measured by resazurin reduction-based assays after 5 days (n=2). Synergy was assessed according to Bliss and visualized by Combenefit.

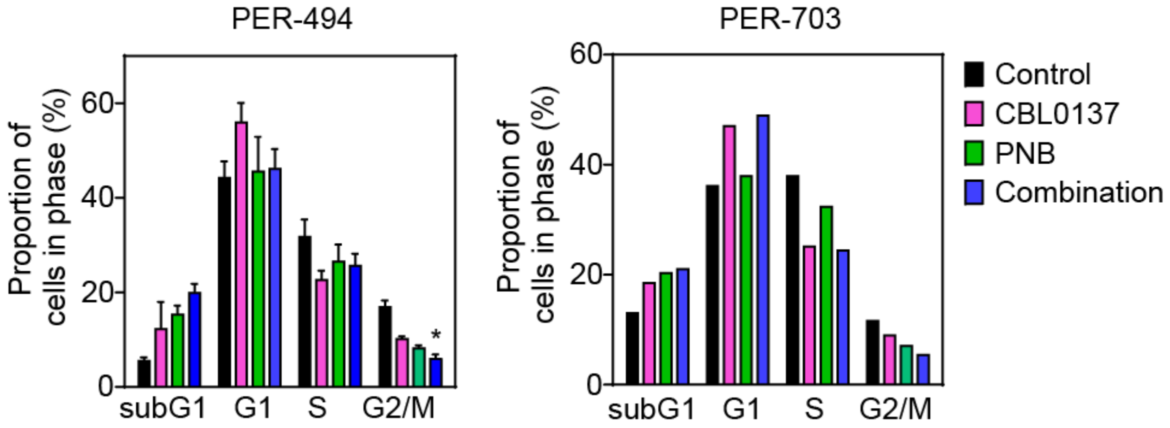

Supplementary Figure 7. The combination of CBL0137 and panobinostat (PNB) has a minor effect on KMT2A-r leukemia cell cycle progression**.** Quantification of cell cycle phase distribution of PER-494 (n=2) and PER-703 KMT2A-r leukemia cells measured by flow cytometry through propidium iodide staining. Cells were treated with CBL0137 and PNB either as single agents or the combination for 24 hours. Statistical significance of differences in proportion of cells in a certain cell cycle phase between treatment groups was determined by ANOVA followed by Tukey's multiple comparison test. Asterisks represent significance levels of P values: *, P<0.05. Statistical data displayed at the top of a bar in the graphs correspond to comparisons with control cells.

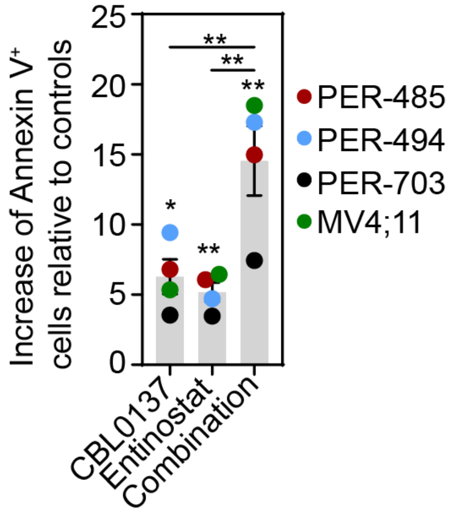

Supplementary Figure 8. The combination of CBL0137 and entinostat induces apoptosis in KMT2A-r leukemia cells. Mean percentage increase in annexin V^+ PER-485, PER-494 and PER-703 cells (includes annexin V^+/7AAD$^-$ and annexin V^+/7AAD$^+$ cells) relative to vehicle-treated cells after treatment with CBL0137 (PER-485: 0.2 µM; PER-494: 0.3 µM; PER-703: 0.3 µM; MV4;11: 0.3 µM), entinostat (PER-485: 250 nM; PER-494: 200 nM; PER-703: 250 nM; MV4;11: 250 nM) or the combination for 48 hours. The significance of the difference in mean percentage increase of annexin V^+ cells after drug treatment versus vehicle treatment was determined by One sample t-tests. For significance determination between drug treatment groups, paired t-tests were used. Asterisks represent significance levels of P values: *, P<0.05; **, P<0.01. Statistical data displayed at the top of a bar in the graphs correspond to comparisons with control cells.

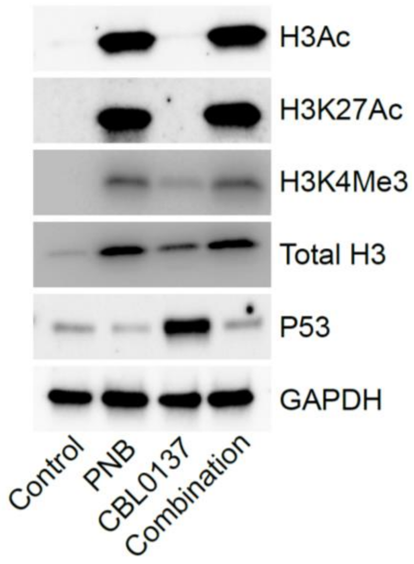

Supplementary Figure 9. The combination of CBL0137 and panobinostat (PNB) does not significantly alter acetylation or methylation levels on KMT2A-r associated histone marks compared to PNB alone. Western blots showing expression of histone marks and P53 in PER-485 cells treated with either 0.3 µM CBL0137, 10 nM PNB, or the combination of the two drugs for 24 hours. Images are representative of two independent experiments.

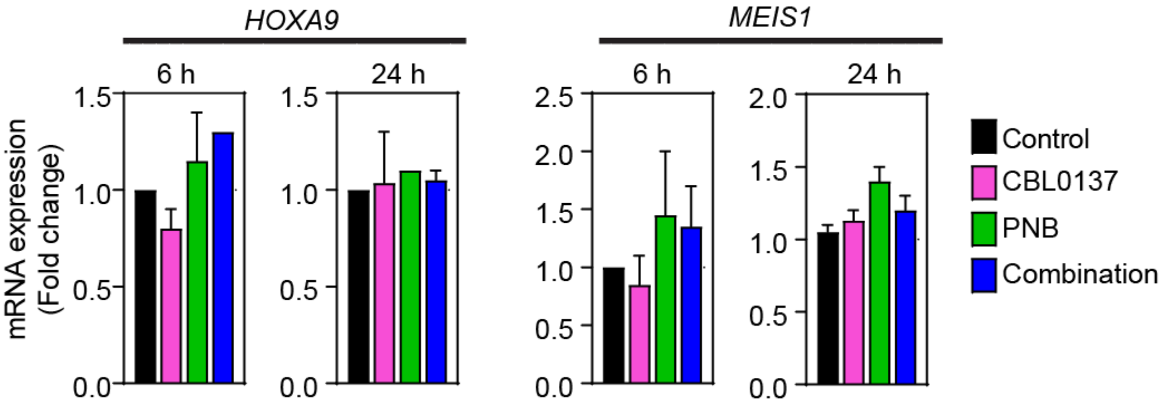

Supplementary Figure 10. The combination of CBL0137 and panobinostat (PNB) does not significantly alter *MEIS1* and *HOXA9* expression levels. Gene expression in PER-485 cells treated with 0.3 µM CBL0137, 10 nM PNB or the combination for 6 or 24 hours relative to vehicle-treated control cells as determined by qRT-PCR.

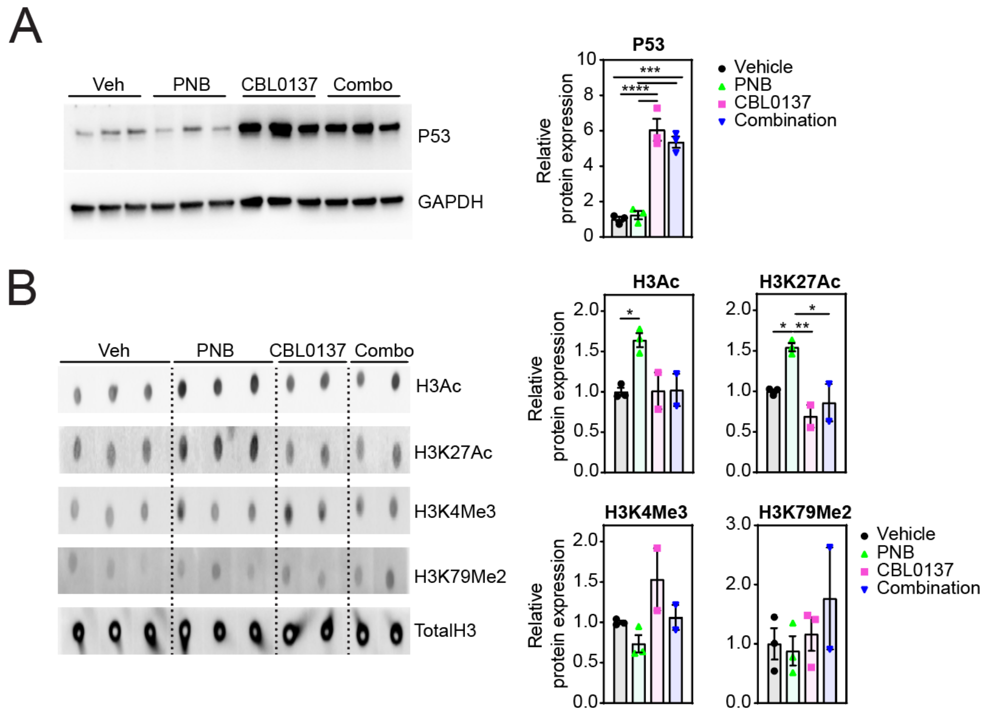

Supplementary Figure 11. (A) Western blots showing expression of P53 in splenocytes harvested from KMT2A-r ALL PDX-engrafted mice treated with either panobinostat (PNB), CBL0137, or the combination of the two drugs. Densitometric quantification of the Western blots is shown in the bar graph on the right. **(B)** Dot blots showing levels of specific histone modifications in histones isolated from splenocytes harvested from KMT2A-r ALL PDX-engrafted mice treated with PNB, CBL0137, or the combination of the two drugs. Densitometric quantification of the Western blots is shown in the bar graph on the right. The significance of the difference in mean ± SE is determined by one-way ANOVA followed by Tukey's multiple comparison test. Asterisks represent significance levels of P values: *, P<0.05; **, P<0.01, ***, P<0.001, ****, P<0.0001.

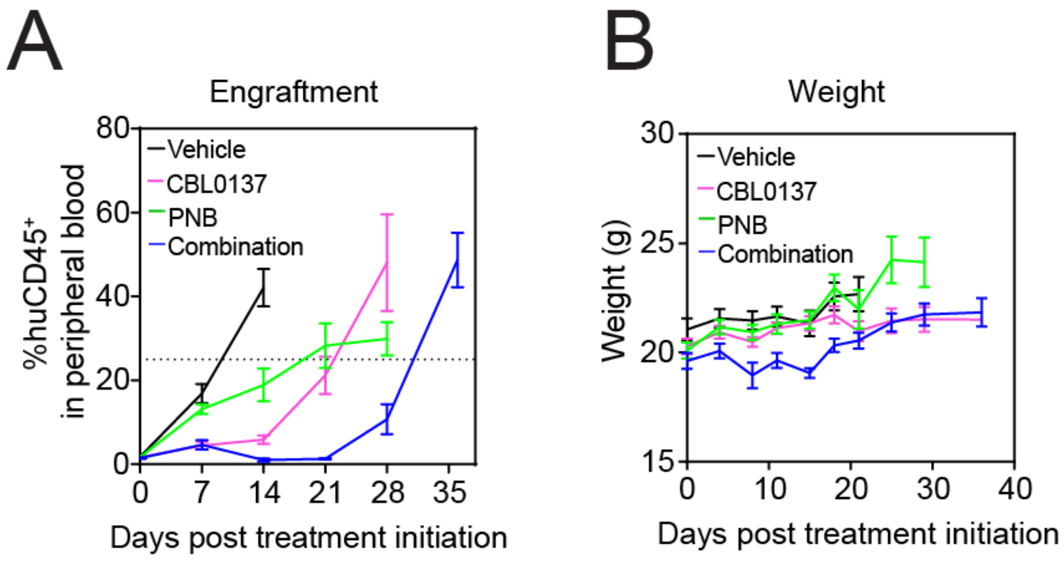

Supplementary Figure 12. The combination of CBL0137 and panobinostat (PNB) inhibits progression in an infant KMT2A-r ALL patient derived xenograft model. (A) Leukemia cell levels as measured by enumeration of the % huCD45^{+} cells in the peripheral blood over time. Graph shows the mean %huCD45^{+} cells in peripheral blood per group ± SE of the individual mice shown in Fig. 3B. **(B)** Evolution of mean weight per group ± SE.
